# Insight into biomass feedstock on formation of biochar-bound environmentally persistent free radicals under different pyrolysis temperatures†

**DOI:** 10.1039/d2ra03052g

**Published:** 2022-07-04

**Authors:** Yu Wang, Xinfeng Gu, Yue Huang, Zhuhong Ding, Yijun Chen, Xin Hu

**Affiliations:** School of Environmental Science & Engineering, Nanjing Tech University 30 Puzhu Southern Road Nanjing 211816 PR China dzhuhong@njtech.edu.cn; State Key Laboratory of Analytical Chemistry for Life Science, Centre of Materials Analysis and School of Chemistry & Chemical Engineering, Nanjing University 22 Hankou Road Nanjing 210023 PR China

## Abstract

Environmentally persistent free radicals (EPFRs) in biochars have the ability of catalytic formation of reactive oxygen species, which may pose potential oxidative stresses to eco-environment and human health. Therefore, comprehending the formation and characteristics of EPFRs in biochars is important for their further applications. In this study, the woody lignocellulosic biomass (wood chips, pine needle and barks), non-woody lignocellulosic biomass (rice husk, corn stover, and duckweed), and non-lignocellulosic biomass (anaerobically digested sludge) were selected as biomass feedstock to prepare biochars under different pyrolysis temperatures (200–700 °C). The impact of biomass feedstock on formation of biochar-bound EPFRs was systematically compared. Elemental compositions and atomic ratios of H/C and O/C varied greatly among different biomass feedstocks and the subsequently resulting biochars. EPFRs in biochars derived from the studied lignocellulosic biomass have similar levels of spin concentrations (10^18^–10^19^ spins per g) except for lower EPFRs in biochars under 200 and 700 °C; however, sludge-based biochars, a typical non-lignocellulosic-biomass-based biochar, have much lower EPFRs (10^16^ spins per g) than lignocellulosic-biomass-based biochars under all the studied pyrolysis temperatures. Values of *g* factors ranged from 2.0025 to 2.0042 and line width was in the range of 2.15–11.3 for EPFRs in the resulting biochars. Spin concentrations of biochar-bound EPFRs increased with the increasing pyrolysis temperatures from 200 to 500 °C, and then decreased rapidly from 500 to 700 °C and oxygen-centered radicals shifted to carbon-centered radicals with the increasing pyrolysis temperatures from 200 to 700 °C for all the studied biomass feedstock. 300–500 °C was the appropriate pyrolysis temperature range for higher levels of spin concentrations of biochar-bound EPFRs. Moreover, EPFRs' concentrations had significantly positive correlation with C contents and weak or none correlation with contents of transition metals. Overall, different types of biomass feedstock have significant impact on the formation of EPFRs in the resulting biochars.

## Abstract 2

Environmentally persistent free radicals (EPFRs) in biochars have the ability of catalytic formation of reactive oxygen species, which may pose potential oxidative stresses to eco-environment and human health.

## Graphical abstract

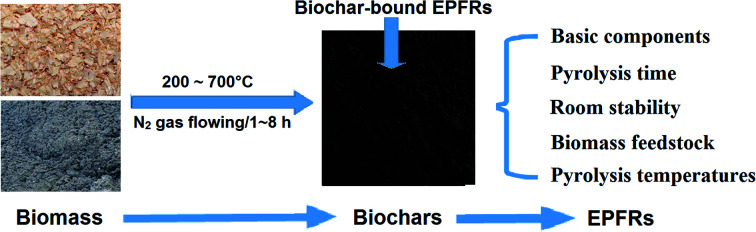

## 1. Introduction

Free radicals are involved in many processes in chemistry, environmental science, life sciences, and so on. Some free radicals have short lifetimes of only a fraction of a second and are therefore called transient radicals or unstable radicals; while other free radicals can exist for minutes or up to several months and years, and are called as persistent free radicals (PFRs) or stable free radicals.^1–3^ PFRs are found in many environmental matrices such as atmospheric particulate matter, fly ash, and biochar, which are generally generated from combustion and thermal processing of organic materials, so PFRs in environmental matrices are called environmentally persistent free radicals (EPFRs).^1–4^ Free radicals contain one or more unpaired electrons, which results in high chemical reactivity. So EPFRs can induce the formation of reactive oxygen species (ROS) such as superoxide radical (O_2_˙^−^) and hydroxyl radical (˙OH), commonly associated with aging, cell damage, and possibly some diseases.^5,6^ Therefore, EPFRs may pose a potential risk to the ecosystem and human health *via* the oxidation stress and are considered as emerging contaminants.^3^

Biochar is a carbon-rich organic–inorganic composite derived from the slow pyrolysis of biomass feedstock, such as wood, straw, manure, or biosolid, under oxygen-limited conditions and has been widely used in the carbon sequestration, wastewater treatment and soil amendment/improvement.^7–9^ The previous investigations reveal that biochars contain abundant EPFRs.^4,10^ The inhibition of seed germination and growth of root and shoot by biochar-bound EPFRs was reported.^11^ So more investigations on formation and characteristics of biochar-bound EPFRs should be carried out before their soil applications.

Biomass feedstock is one of the key factors for the physical and chemical properties of the as-prepared biochars.^12^ The considerable variation in cellulose and lignin contents among biomass feedstock may be important reason for the differences on the physical and chemical properties of the as-prepared biochars. Now biochars have been successfully prepared from various biomass feedstock from the forestry, agriculture, sludge of municipal sewage treatment plants, and food industry.^12^ These biomass feedstock can be categorized into lignocellulosic biomass and non-lignocellulosic biomass according to cellulose and lignin contents, or woody and non-woody biomass according to lignin contents. Residues from forestry and trees are primary sources of woody lignocellulosic biomass while agricultural crops and residues, animal waste, urban and industrial solid waste are common sources of non-woody lignocellulosic biomass.^13^ The woody biomass has characteristics of low ash and moisture, high calorific value and bulk density, and less voidage while high ash and moisture, low calorific value and bulk density, and higher voidage for the non-woody biomass were reported.^13^ Municipal sludge and alga residues are the typical non-woody non-lignocellulosic biomass. Characteristics and levels of EPFRs have been reported in biochars derived from coconut shell, eucalyptus leaves, walnut shell, pine needles, sawdust of poplar and pine, cow manure, peanut shells, corn stalk, rice husk, wheat straw and maize straw.^14–18^ These are of woody and non-woody lignocellulosic biomass mainly (*i.e.* woody plants and agricultural residues). Relevant literature data of types of biomass, pyrolysis parameters (pyrolysis temperature, and residence time), EPFRs' characteristics (*g*-factor, line width, and spin concentrations of EPFRs) were reviewed (Table S1†). Compared to EPFRs in biochars derived from lignocellulosic biochars (Table S1†), data in biochars derived from non-lignocellulosic biomass are very limited.^19–21^ Sludge is a typical non-lignocellulosic biomass and sludge-based biochars receive widely attention in soil remediation, wastewater treatment, chemical catalysis, and so on,^22,23^ but there were limited investigations on EPFRs in sludge-based biochars.^24^ For example, the *g*-values of PFRs in sludge-based pyro-biochars were reported, but the spin concentrations of PFRs were not offered.^25–27^ Therefore, formation and characteristics of EPFRs in non-lignocellulosic biomass should be comparison further.

In the present study, woody lignocellulosic biomass (wood chips, pine needle and barks), non-woody lignocellulosic biomass (rice husk, corn stover, and duckweed), and non-lignocellulosic biomass (anaerobically digested sludge) were selected as biomass feedstock to prepare biochars through the slow pyrolysis under oxygen-limited atmosphere. The comparisons of biomass feedstock on formation of biochar-bound environment persistent free under different pyrolysis temperatures were investigated. This will be beneficial for the evaluation of the positive or negative impact of biochar due to biochar-bound EPFRs before their large-scale application in environment.

## 2. Materials and methods

### 2.1. Materials and reagent

Reagents of analytic grade were purchased from Nanjing Chemical Reagent Co. 18.2 MΩ cm of deionized (DI) water was used in the all experiments.

### 2.2. Preparation of biochars and component analyses of the biomass and the resulting biochars

The selected biomass feedstock in this study included woody lignocellulosic biomass (wood chips, pine needle and barks), non-woody lignocellulosic biomass (rice husk, corn stover, and duckweed), and non-lignocellulosic biomass (anaerobically digested sludge). These are commonly and easily available in China. All air-dried biomass was ground into powder by using a knife mill. The powders were placed inside quartz tube of a tubular furnace and then heated to the designed pyrolysis temperature of 200, 300, 400, 500, 600 and 700 °C for 1–8 h under N_2_ gas flowing for oxygen-limited conditions. The pristine biochars were placed in zipper bags for further analysis (details can be seen in our previous reports).^28^ Biochars derived from rice husk, wood chips, pine needle, anaerobically digested sludge, corn stover, barks, and duckweed were noted as RH*x*, WC*x*, PN*x*, ADS*x*, CS*x*, BK*x*, and DW*x* (“*x*” referred to as the pyrolysis temperature of 200, 300, 400, 500, 600, and 700 °C), respectively. The resulting biochars can be classified as non-woody-lignocellulosic-biomass-, woody-lignocellulosic-biomass-, and non-lignocellulosic-biomass-based biochars. They were stored in zip-lock polyethylene bags for further treatment. Biomass feedstock of rice husk, wood chips, pine needle, anaerobically digested sludge, corn stover, barks, and duckweed were marked as RH, WC, PN, ADS, CS, BK, and DW, respectively.

Contents of C, H, and N in the biomass feedstock and the resulting biochars were analyzed by using a CHN Elemental Analyzer (Carlo-Erba NA-1500) and the contents of O were the differences between 100% (the total fraction) and the sum of fractions of C, H, N, and ash. Ash content was the fraction of ash mass after ashing at 500 °C to its initial mass. Ash was dissolved with diluted *aqua regia* and then transferred to a 50 mL volumetric flask with deionized water. Concentrations of mineral elements in the digestion solution were determined by using an inductively coupled plasma optical emission spectroscopy (ICP-OES, PerkinElmer Optima 5300 DV, USA). When the relative deviation of parallel samples was more than 10%, the experiments were done again.

### 2.3. Radicals in the resulting biochars

The resulting biochar samplings (10.00 mg) were put into quartz tubes, respectively, and then placed in an X-band EPR spectrometer (EMX 10/12; Bruker). EPFRs in all samplings were recorded with dual cavity mode of EPR. Parameters for EPR analyses were seen in our previous report.^20^ Briefly, they were center field (3480 G), sweep width (200 G), microwave power (0.499 mW), modulation amplitude (0.50 G), modulation frequency (100 kHz), time constant (40.96 ms), and sweep time (83.89 s). A self-made coal sample with 2.64 × 10^14^ spins^29^ is used as an external standard for the quantitative analysis. The spins of samplings can be obtained due to the ratio of spin number equal to the ratio of quadratic integral value. All EPR signals presented in this study are average values of triplicate samplings.

To investigate the effects of the pyrolysis time on biochar-bound EPFRs, EPR signals of rice husk-based biochars of the pyrolysis temperature of 400 °C (according to the pre-experimental results) at the pyrolysis time of 1, 2, 4, and 8 h, respectively, were recorded.

To investigate the effects of the pyrolysis temperatures on biochar-bound EPFRs, EPR signals of rice-husk-, wood-chip-, pine-needle-, anaerobically-digested-sludge-based biochars under the pyrolysis temperature of 200, 300, 400, 500, 600 and 700 °C at the pyrolysis time of 4 h, respectively, were recorded.

To investigate the effects of the biomass feedstock on biochar-bound EPFRs, EPR signals of biochars derived from rice husk, wood chips, pine needle, anaerobically digested sludge, corn stover, barks, and duckweed under the pyrolysis temperature of 500 °C at the pyrolysis time of 4 h, respectively, were recorded.

To identify the room stability of EPFRs in samplings stored at room temperature, EPR signals of rice husk-based biochars of the pyrolysis temperature of 200–700 °C were re-determined after 7 and 18 days, respectively.

### 2.4. Data analysis

All of the values were the mean of three replicates. The descriptive statistics and Pearson correlation analysis between contents of transition metals and spin concentrations of EPFRs in biochars were carried out by using version 18 of the SPSS for Windows software package (SPSS Inc., Chicago, IL). Figures were drawn by using Origin 8.0.

## 3. Results and discussion

### 3.1. Components of the biomass and the resulting biochars

Basic chemical compositions and atomic ratios of the biomass feedstock were listed in Table 1. Table 1 shows that their chemical compositions of C, H, O, N, and ash (mineral elements) varied greatly. For example, ADS has the highest contents of ash (mineral elements) and lowest of C, H, and O. PN has the highest contents of H and C while WC shows the highest contents of O and lowest of N. Atomic ratios of H/C and O/C of ADS was obviously higher than those of the others (Table 1). These suggest the differences of the biomass feedstock of non-woody lignocellulosic plant, woody plant, and sludge. Coefficients of variation were 61.8% for N, 25.8% for C, 15.0% for H, 106% for ash, 14.1% for O, 18.3% for H/C, and 31.3% for O/C, respectively, suggesting that contents of N and ash (mineral elements) among those biomass feedstock differed greatly.

**Table:** Basic elemental compositions (%) and atomic ratios of the biomass feedstock

	N	C	H	Ash	O	H/C	O/C
BK	1.72	45.1	5.75	11.9	35.5	1.53	0.59
PN	4.24	49.4	6.42	3.26	36.7	1.56	0.56
DW	2.63	37.0	5.71	8.02	46.6	1.85	0.95
CS	1.64	42.4	6.09	9.17	40.7	1.72	0.72
ADS	3.85	18.6	3.87	41.2	32.5	2.50	1.31
RH	1.05	36.9	5.51	12.4	44.1	1.79	0.90
WC	0.590	44.7	6.24	1.54	46.9	1.68	0.79
Min	0.59	18.6	3.87	1.54	32.5	1.53	0.56
Max	4.24	49.4	6.42	41.2	46.9	2.50	1.31
Average	2.25	39.2	5.66	12.5	40.4	1.80	0.83
SD	1.39	10.1	0.85	13.3	5.7	0.33	0.26

The contents of C, H, O, N, ash and main mineral elements of the resulting biochars were listed in Table S2.† Table S2† shows that with the increasing pyrolysis temperature from 200 to 700 °C contents of C and ash of WC*x*, ADS*x*, RH*x*, and PN*x* increased while H, N and O contents decreased, and atomic ratios of H/C and O/C also decreased. Table S2† also shows that the main mineral elements' contents varied greatly among WC*x*, ADS*x*, RH*x*, and PN*x*, derived from different biomass feedstock (Table S2†). These were consistent with the previous reports.^30^ Generally, ADS*x* had the highest contents of ash (mineral elements); contents of mineral elements of ADS*x* such as Al, Ca, Na, Cu, Fe, Mn, and Zn were significant higher than those of WC*x*, RH*x*, and PN*x* (Table S2†). C contents of ADS*x* were significant lower than those of WC*x*, RH*x*, and PN*x* (Table S2†). These were consistent with chemical compositions of the biomass feedstock (Table 1). Atomic ratios of H/C and O/C in the resulting biochars (Table 1) were generally higher than those of the corresponding biomass feedstock (Table 1). It is believed that the levels of carbonization and high aromaticity of biochars were negatively related to H/C ratios^31–33^ while O/C atomic ratios reflect the nonpolarity and hydrophilicity.^34^ So the aromaticity and hydrophilicity of the resulting biochars differed greatly and impacted by the biomass feedstock and the pyrolysis temperatures (Table 1).

### 3.2. Effects of pyrolysis time on EPFRs in the resulting biochars

**Fig. 1:**
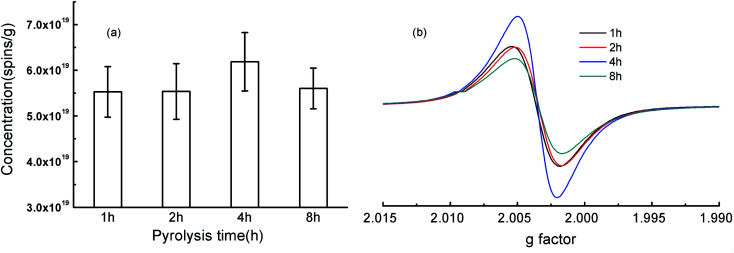
Concentrations (spins per g) and g factor of the rice-husk-based biochars under the pyrolysis temperature of 400 °C and the pyrolysis time of 1, 2, 4, and 8 h.

According to the related literature, the pyrolysis time for biochars were generally about 2–4 h.^35,36^ In this study, the rice-husk-based biochars at the pyrolysis time of 1, 2, 4, and 8 h were prepared and spin concentrations (spins per g) and *g* factor of biochar-bound EPFRs were analyzed (Fig. 1, detail data seen in Table S3†). Line width (Δ*H*_p–p_) of this study (Table S3†) was in the range of those in the literature data (Table S1†). Ranges of EPFRs' concentrations were 5.53–6.19 × 10^19^ spins per g (Fig. 1a). It seems that EPFRs' concentrations increased slightly from 1 to 4 h and then decreased slightly from 4 to 8 h (Fig. 1a). However, they were in the same order of magnitude, and no significant differences were found on the spin concentrations (spins per g) of biochar-bound EPFRs (Fig. 1a). This was consistent with the previous reports that the heating time had no significant effect on properties of the resulting biochar.^36^ For example, there were no significant changes on spin concentration of EPFRs of biochars derived from cellulose–urea mixture under the pyrolysis temperatures of 500 °C when the residence times were 5, 10, 15, 20 and 30 min, while they increased significantly from 1 to 5 min.^37^ Biochar pyrolyzed for 1–5 h shows that the relative concentration of EPFRs gradually increased, arrived maximum values at 4 h, and decreased at 5 h.^17^

Generally, *g* factor is used to classify an unpaired electron in a different chemical environment.^2^ It is generally accepted that *g* factors of <2.0030 is of carbon-centered radicals, *g* factors in the range of 2.0030–2.0040 is of carbon-centered radicals with oxygen atoms, and *g* factors of >2.0040 is of oxygen-centered radicals.^4,10,38^ Values of *g* factor ranged from 2.0026 to 2.0029 (Fig. 1b). So the type of EPFRs in RH*x* were mainly belonged to the carbon-centered radicals with oxygen atoms and no changes on the type of EPFRs were observed (Fig. 1b). These suggesting that the pyrolysis time is not the key factor for the concentrations and the type of EPFRs.

### 3.3. Stability of EPFRs in the resulting biochars under room temperature

**Fig. 2:**
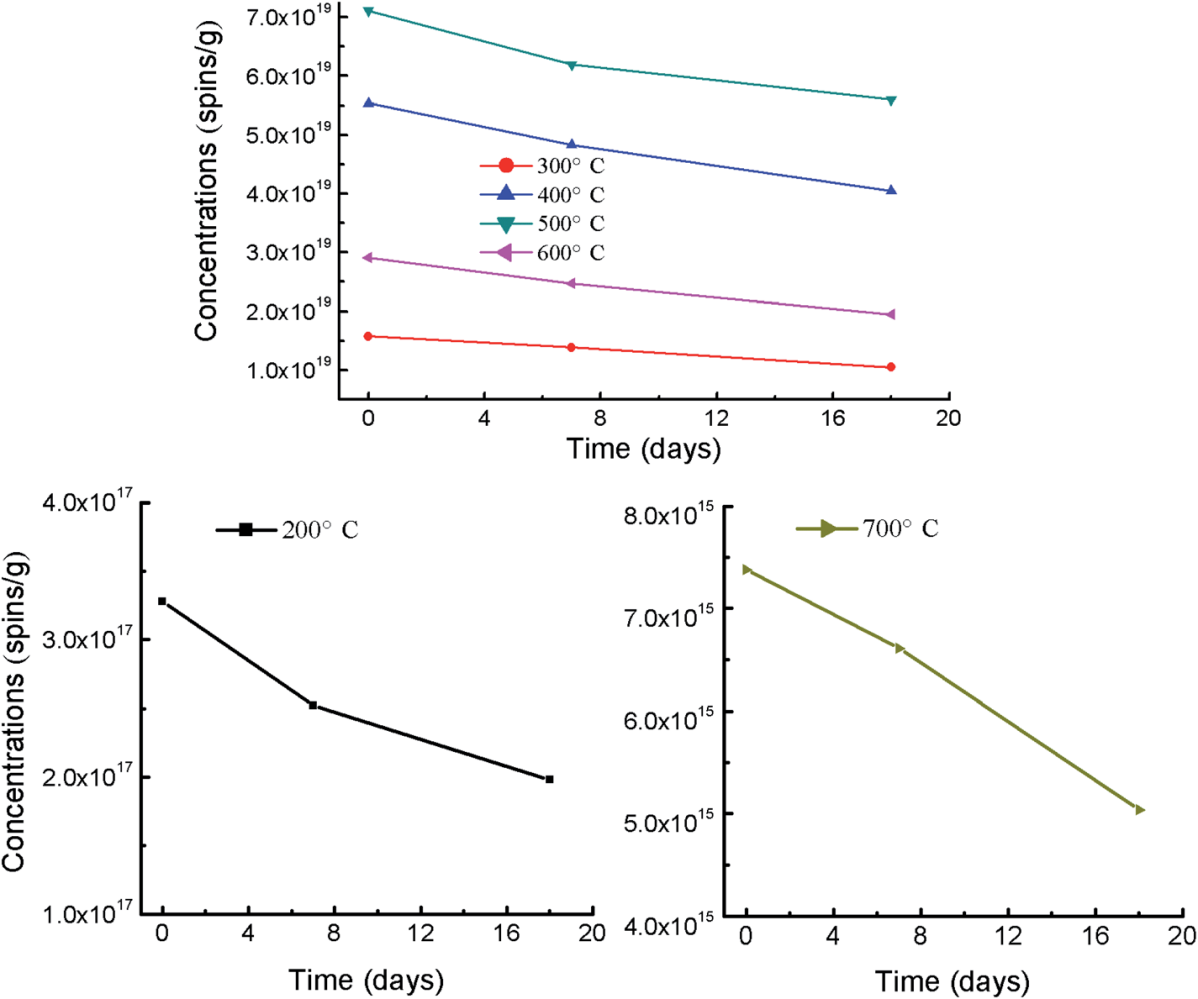
Decay of spin concentrations of EPFRs in RH*x* under room temperature.

Stability of EPFRs is an important parameter for environmental transfer and risks of biochar-bound EPFRs. Therefore, the decay of EPFRs in biochars stored under room temperature was investigated (Fig. 2, detail data seen in Table S4†). EPFRs in RH*x* were analyzed in the 7th and 18th day of after the initial analysis (Table S4†). Compared to the initial spin concentrations, EPFRs' concentrations in 7th/18th day reduces by 23.2/39.6, 12.0/33.5 12.8/26.9, 12.9/21.2, 15.1/33.0, and 10.4/31.7% for 200 (RH200), 300 (RH300), 400 (RH400), 500 (RH500), 600 (RH600), and 700 °C (RH700), respectively (Fig. 2). The average reduction was 14.4 and 31.0% for 7th and 18th day. These shows that EPFRs' concentrations in RH*x* decreased slowly with the increasing storage time under room temperature. The decay of EPFRs has been reported in the previous lab-simulating studies.^39,40^ The previous literature shows that EPFRs can reacted with O_2_ to form superoxide radical (O_2_˙^−^), and finally hydroxyl radical (˙OH).^40^ Therefore, biochar-bound EPFRs exposed to ambient oxygen may form transient free radicals of reactive oxygen species. This may be the mechanism of the decay of EPFRs.

Values of *g* factor ranged from 2.0025 to 2.0035 for the initial analysis, from 2.0019 to 2.0036 for the 7th day, and from 2.0024 to 2.0039 for the 18th day in this study (Table S4†). So the type of biochar-bound EPFRs were mainly belonged to carbon-centered radicals and carbon-centered radicals with oxygen atoms. A slight variation of *g* values among the initial analysis, 7th day and 18th day, however, the types of EPFRs in RH*x* didn't change with the increasing storage time under room temperature. Overall, the increasing storage time under room temperature decrease the EPFRs' concentrations.

### 3.4. Effects of pyrolysis temperature on EPFRs in the resulting biochars

Spin concentrations and *g* factor of RH*x*, ADS*x*, WC*x*, and PN*x* were listed in Table 2. The maximum EPFRs' concentrations were observed at the pyrolysis temperatures of 400 °C for ADS*x* and WC*x* and 500 °C for RH*x* and PN*x* (Table 2). The minimum EPFRs' concentrations were observed at the pyrolysis temperatures of 700 °C for ADS*x*, WC*x*, RH*x* and PN*x* (Table 2). The ratio of the highest to lowest spin concentration for biochars of same biomass feedstock pyrolyzed at 200, 300, 400, 500, 600 and 700 °C was 9634 for RH*x*, 150 for ADS*x*, 381 657 for WC*x*, and 93 652 for PN*x*, suggesting the great variations of EPFRs' concentrations impacted by the pyrolysis temperatures. EPFRs' concentrations increased with the increasing pyrolysis temperatures from 200 to 400 °C for ADS*x* and WC*x* and from 200 to 500 °C for RH*x* and PN*x* and then decreased greatly (Table 2). 300–500 °C may be the reasonable range of the pyrolysis temperatures for EPFRs in the resulting biochars (Table 2). So EPFRs' levels in the resulting biochars derived from woody and lignocellulosic biomass (wood chips, pine needle and barks), non-woody and lignocellulosic biomass (rice husk, corn stover, and duckweed), and non-woody and non-lignocellulosic biomass (anaerobically digested sludge) had similar trends with the increasing pyrolysis temperatures. Table S1† also shows that the medium pyrolysis temperatures are beneficial for the formation of EPFRs in non-lignocellulosic and lignocellulosic biomass-based biochar.

Spin concentrations (spins per g), *g* factor, and line width (Δ*H*p–p) of the resulting biochars derived from four feedstock under different pyrolysis temperatures (200–700 °C)

**Table:** 

	RH*x*			ADS*x*		
	Spins per g	*g*-Factor	Δ*H*p–p	Spins per g	*g*-Factor	Δ*H*p–p
200 °C	3.28 × 10^17^	2.0035	6.45	6.92 × 10^17^	2.0036	7.82
300 °C	1.58 × 10^19^	2.0033	7.04	6.97 × 10^17^	2.0033	9.78
400 °C	5.54 × 10^19^	2.0028	5.47	1.15 × 10^18^	2.0030	10.8
500 °C	7.11 × 10^19^	2.0030	4.30	2.44 × 10^17^	2.0027	11.3
600 °C	2.91 × 10^19^	2.0025	3.71	2.51 × 10^16^	2.0026	10.2
700 °C	7.38 × 10^15^	2.0025	2.15	7.66 × 10^15^	2.0025	8.60

**Table:** 

	WC*x*			PN*x*		
	Spins per g	*g*-Factor	Δ*H*p–p	Spins per g	*g*-Factor	Δ*H*p–p
200 °C	4.63 × 10^17^	2.0037	5.08	2.00 × 10^18^	2.0036	6.84
300 °C	2.32 × 10^19^	2.0042	6.84	2.34 × 10^19^	2.0038	8.60
400 °C	6.45 × 10^19^	2.0033	4.69	4.41 × 10^19^	2.0031	4.30
500 °C	5.86 × 10^19^	2.0034	5.28	9.00 × 10^19^	2.0033	4.69
600 °C	2.69 × 10^19^	2.0034	8.02	9.73 × 10^19^	2.0029	4.11
700 °C	1.69 × 10^14^	2.0028	7.23	9.61 × 10^14^	2.0029	5.28

Table 2 shows that values of *g* factor were 2.0035–2.0025 for RH*x*, 2.0033–2.0025 for ADS*x*, 2.0045–2.0028 for WC*x*, and 2.0038–2.0029 for PN*x*. Generally, *g* values decreased with the increasing pyrolysis temperatures from 200 to 700 °C. Types of EPFRs were generally belonged to the carbon-centered radicals with oxygen atoms for the biochars of 200 °C, and then shifted to carbon-centered radicals with the increasing pyrolysis temperatures from 200 to 700 °C as the classification criteria mention above.^38,41^ These were consistent with the previous reports (Table S1†). So the pyrolysis temperatures is an important factor on EPFRs in the resulting biochars, which impacts not only EPFRs' concentrations but also EPFRs' types.

It was reported that the pyrolysis temperature of 300–700 °C had great influence on the concentrations and types of EPFRs in biochars derived from pine needles, wheat straw, maize straw and so on.^14,15,17,18^ Values of *g* factor decrease with the increasing pyrolysis temperatures, and oxygen-centered radicals are predominate in biochars at low pyrolysis temperatures while the carbon-centered radicals prevalent at higher pyrolysis temperatures.^11,15,17,18^ The similar results were observed by Yang *et al.* (2016), and Qin *et al.* (2016 & 2017).^16,42,43^ Odinga *et al.* (2020) reviewed that little or no free radicals were generated in biochar at the pyrolysis temperatures of ≤200 °C; oxygen- and carbon-centered radicals with oxygen atoms radicals were produced in biochars at ∼300 °C to ∼500 °C; free radicals in biochar were decreased drastically at the pyrolysis temperatures of ∼500 °C to ∼700 °C; and little to no EPFRs would be formed at the pyrolysis temperatures of >700 °C.^4^ The depolymerization, fragmentation and restructuring of lignin, cellulose, and hemicellulose in biomass feedstock at about 200–500 °C generally produce phenol or quinone moieties during the slow pyrolysis, and then these moieties would be decomposed and shift to amorphous phase and crystalline structures of graphitic carbon with the increasing temperatures.^4^ It is believed that biochar-bound EPFRs vary with the formation and decomposition of the phenol or quinone moieties. In a word, the increasing pyrolysis temperatures had similar impact on EPFRs' levels and types in the resulting biochars derived from different biomass feedstock (woody lignocellulosic biomass, non-woody lignocellulosic biomass, and non-lignocellulosic biomass).

### 3.5. Effects of biomass feedstock on EPFRs in the resulting biochars

**Fig. 3:**
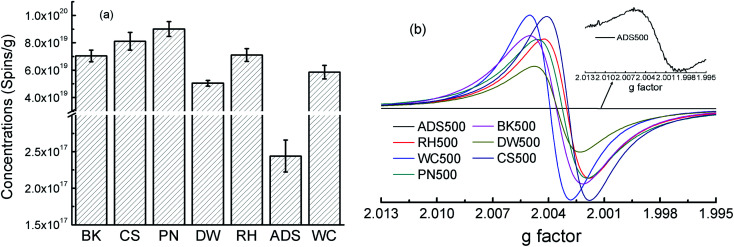
Concentrations (spins per g) and g factor of the resulting biochars derived from seven feedstock under the pyrolysis temperature of 500 °C.

Fig. 3a shows that EPFRs' concentrations differed greatly among BK*x*, CS*x*, PN*x*, DW*x*, RH*x*, ADS*x*, and WC*x* at the pyrolysis temperatures of 500 °C. For example, EPFRs' concentration of BK500, CS500, PN500, DW500, RH500, and WC500 was in the same magnitude (∼10^19^ spins per g) and about two orders of magnitude higher than that of ADS500 (10^17^ spins per g) (Fig. 3a). Fig. 3b shows that types of EPFRs in BK500, CS500, PN500, RH500, DW500, and WC500 were belonged to the carbon-centered radicals with oxygen atoms while that of ADS500 was of carbon-centered radicals. Overall, concentrations and types of EPFRs in sludge-based biochars differed greatly from those in the lignocellulosic-biomass-based biochars. This was consistent with the previous report that the spin concentration of EPFRs in sludge-based biochar ranged from 2.16 ×  10^16^ to  4.42 ×   10^16^ spins per g in the pyrolysis temperature of 200  to 600 °C.^44^ In some papers, the *g*-factor of PFRs in sludge-based pyro-biochars were reported while their spin concentration were not offered,^25–27^ which cannot be compared with our results. The spin concentration were generally 10^18^–10^19^ spins per g for the lignocellulosic-biomass-based biochars, consistent with our results. Therefore, EPFRs' levels in the biochars derived from lignocellulosic biomass were similar, but significant higher than those from non-lignocellulosic biomass, which might be due to element composition among lignocellulosic and non-lignocellulosic biomass and the resulting biochars (Tables 2 and S2†). Spin concentrations and *g*-values in this study were consistent with the literature data (Table S1†). So sludge-based biochars, a typical non-lignocellulosic biomass, has much lower EPFRs than lignocellulosic-biomass-based biochars although sludge-based biochars have better performance on sorption of contaminants than common pristine biochars derived from lignocellulosic biomass.^22^ Lower EPFRs of sludge-based biochars are favorable to their agricultural application.

### 3.6. Relationship between EPFRs and components of the resulting biochars

Elimination of HCl or H_2_O form phenol or quinone moieties and then electron transfer from those moieties to transition metals to produce EPFRs in the resulting biochar was proposed as the possible formation mechanisms of biochar-bound EPFRs.^4^ This indicates that the phenol or quinone moieties and transition metals is important in the formation of EPFRs. Therefore, Pearson correlation analysis between contents of transition metals and spin concentrations in biochars was carried out (Table 3). Table 3 shows that spin concentrations of biochar-bound EPFRs were significantly positive correlation with contents of C (*p* < 0.01) and none or weak correlation with contents of H, N, O and transition elements (Cu, Fe, Mn, and Zn) (*p* > 0.05), respectively. Total contents of transition elements (the sum of fractions of Cu, Fe, Mn, and Zn) ranged from 38.6 to 47 748 mg kg^−1^ with a median value of 1059 mg kg^−1^. There was also no significant correlation between total contents of transition metals and spin concentrations of biochar-bound EPFRs. Table S2† shows that sludge-based biochars had higher contents of mineral elements, however, sludge-based biochars had much lower EPFRs than lignocellulosic-biomass-based biochars (Table 2). Therefore, all these may suggest that contents of transition metals may be not a key factor in the formation of EPFRs. As mentioned above, the mechanisms of EPFR formation in some previous literature is suggested as the chemisorption of organic precursor molecule, and followed by electron transfer from the adsorbate to the transition metal atom.^10^ However, PFRs (stable free radicals) can be detected when the pyrolysis of lignin and cellulose without the presence of metal(s).^37,45,46^ The pyrolysis of pure cellulose or lignin can form stable organic radicals in the resulting biochars, suggesting that metals are not necessary during the formation of PFRs when the pyrolysis of biomass.^37,45^ For example, Liao *et al.* (2014) reported that the hemolytic cleavage of the chemical bonds in the macromolecules during carbonization promoted EPFR generation in biochar.^11^ Odinga *et al.* reviewed the mechanisms of EPFR formation including the interaction between organic precursor molecule and transition metal, and the breaking of chemical bonds in macromolecules.^4^ The presence of transition metals may be beneficial to stabilize particle-bound free radicals. Therefore, further investigations should be done to elucidate the formation mechanisms of EPFRs, especially comparative investigations on EPFRs formation between sludge-based biochar and lignocellulosic-based biochars.

**Table:** Pearson correlation coefficients among main/trace-transitional elements and EPFRs in the resulting biochars

	Spin	C	H	N	O	Ash	Cu	Fe	Mn	Zn
Spin	1.00	0.44^a^	0.14	−0.064	0.44^a^	−0.006	−0.006	−0.37	−0.24	−0.22
C		1.00	0.33	−0.24	1.00^b^	0.11	0.11	−0.89^b^	−0.47^a^	−0.30
H			1.00	0.23	0.33	0.93^b^	0.93^b^	−0.71^b^	−0.62^b^	−0.71^b^
N				1.00	−0.24	0.11	0.11	0.11	0.43^a^	0.23
O					1.00	0.11	0.11	−0.89^a^	−0.44^a^	−0.30
Ash						1.00	1.00^b^	−0.55^b^	0.72^b^	0.87^b^
Cu							1.00	−0.55^b^	0.72^b^	0.87^b^
Fe								1.00	0.022	−0.14
Mn									1.00	0.94^b^
Zn										1.00

a Correlation is significant at the 0.05 level (2-tailed).

b Correlation is significant at the 0.01 level (2-tailed).

## 4. Conclusion

The woody lignocellulosic biomass (wood chips, pine needle and barks), non-woody lignocellulosic biomass (rice husk, corn stover, and duckweed), and non-lignocellulosic biomass (anaerobically digested sludge) were selected as biomass feedstock and elemental compositions and atomic ratios of H/C and O/C confirmed the great differences among different types of biomass feedstock. The great differences on elemental compositions and atomic ratios of H/C and O/C among the resulting biochars were consistent with these of the raw biomass. The pyrolysis time (*i.e.*, 1, 2, 4, and 8 h) had no significant influence on concentrations and types of EPFRs in rice-husk-based biochar of 400 °C. Concentrations of biochar-bound EPFRs increased with the increasing pyrolysis temperature from 200 to 500 °C and decreased greatly from 500 to 700 °C and the types of EPFRs shifted from the carbon-centered radicals with oxygen atoms to carbon-centered radicals with the increasing pyrolysis temperatures from 200 to 700 °C. The pyrolysis temperatures of 300–500 °C can obtain higher levels of biochar-bound EPFRs of the carbon-centered radicals with oxygen atoms. Concentrations of EPFRs in biochars derived from sludge were significantly lower than those in other biochars under all studied pyrolysis temperatures in this study, suggesting the great impact of biomass feedstock. The average decay of EPFRs' concentrations was 14.4 and 31.0% for 7 and 18 days' intervals in room temperature. Moreover, EPFRs' concentrations had significantly positive correlation with C contents (*p* < 0.01) and weak or none correlation with contents of transition metals (Cu, Fe, Mn, and Zn) (*p* > 0.05) and there were great differences on contents of transition metals and spin concentrations of EPFRs between sludge-based biochars and lignocellulosic-biomass-based biochars, suggesting that contents of transition metals in biochars may be not a key factor in the formation of EPFRs. Overall, sludge-based biochars, a typical non-lignocellulosic biomass, has much lower EPFRs than lignocellulosic-biomass-based biochars, which is benefit for its environmental application with low oxidation risks posed by EPFRs.

## Data availability

Data and material is available for research purpose and for reference.

## Conflicts of interest

The authors declare that they have no competing interests.

## Supplementary Material

RA-012-D2RA03052G-s001
