# Supplementary material for: Insight into biomass feedstock on formation of biochar-bound environmentally persistent free radicals under different pyrolysis temperatures
Source: RSC Adv. 2022 Jul 4;12(30):19318–26. doi: 10.1039/d2ra03052g (PMC9251640; doi:10.1039/d2ra03052g)
Supplement: RA-012-D2RA03052G-s001 [file RA-012-D2RA03052G-s001.pdf]

Electronic Supplementary Material (ESI) for RSC Advances.
This journal is © The Royal Society of Chemistry 2022

Supplementary materials

Insight into biomass feedstock on formation of biochar-bound environment persistent free radicals under different pyrolysis temperatures

Yu Wang[1], Xinfeng Gu[1], Yue Huang[1], Zhuhong Ding[1*], Yijun Chen[2], Xin Hu[2]

[1] School of Environmental Science & Engineering, Nanjing Tech University, 30 Puzhu Southern Road, Nanjing 211816, P.R. China

[2] State Key Laboratory of Analytical Chemistry for Life Science, Centre of Materials Analysis and School of Chemistry & Chemical Engineering, Nanjing University, 22 Hankou Road, Nanjing 210023, PR China

* Corresponding authors: Email: dzhuhong@njtech.edu.cn (Zhuhong Ding)

Table S1. Relevant literature data of types of biomass, pyrolysis temperature, residence time, g-factor, line width, and spin concentrations of EPFRs

Biomass	Temp/resid * (°C/h)	g-factor	Line width (ΔHp-p)	EPFR Conc. (10^{18} spins/g)	EPFR type(s)	References
**Lignocellulosic biomass (woody biomass)**						
Poplar sawdust	300/1~5	2.0031 ± 0.0002		1.75		1
	400/1~5	2.0027 ± 0.0001		4.17	Carbon-centered	
	500/1~5	2.0026 ± 0.0002		6.23	Carbon-centered	
	600/1~5	2.0024 ± 0.0001		2.48	Carbon-centered	
Pine sawdust	300/1~5	2.0033 ± 0.0003		2.36	Carbon/Oxygen**	1
	400/1~5	2.0029 ± 0.0001		2.23	Carbon-centered	
	500/1~5	2.0028 ± 0.0001		6.44	Carbon-centered	
	600/1~5	2.0027 ± 0.0002		2.09	Carbon-centered	
Pine needles	300/6	2.0044 ± 0.0002	7.2 ± 0.1	5.38 ± 0.02	Oxygen-centered	2
	400/6	2.0037 ± 0.0001	6.8 ± 0.2	15.2 ± 0.03	Carbon/Oxygen	
	500/6	2.0032 ± 0.0001	6.5 ± 0.2	22.3 ± 0.04	Carbon/Oxygen	
	350/na***	2.0034 ± 0.0001	6.6 ± 0.1	1.96 ± 0.01	Carbon/Oxygen	3
	550/na	2.0028 ± 0.0002	4.5 ± 0.2	13.7 ± 0.06	Carbon/Oxygen	
Coconut shell	300/na	2.0041		3.57	Oxygen-centered	4
	400/na	2.0032		11.3	Carbon/Oxygen	
	500/na	2.0026		14.6	Carbon-centered	
	600/na	2.0024		2.74	Carbon-centered	
Walnut shell	300/na	2.0043		1.25	Oxygen-centered	4
	400/na	2.0039		7.29	Carbon/Oxygen	
	500/na	2.0027		28.8	Carbon-centered	
	600/na	2.0026		8.44	Carbon-centered	
Eucalyptus leaves	300/na	2.0043		4.52	Oxygen-centered	
	400/na	2.0039		3.96	Carbon/Oxygen	4
	500/na	2.0027		35.3	Carbon-centered	
	600/na	2.0026		8.77	Carbon-centered	
**Lignocellulosic biomass (non-woody biomass)**						
Wheat straw	300/na	2.0036 ± 0.0001	6.5 ± 0.1	7.72 ± 0.05	Carbon/Oxygen	3
	400/na	2.0030 ± 0.0002	5.0 ± 0.2	16.5 ± 0.09	Carbon/Oxygen	

	500/na	2.0029 ± 0.0001	4.8 ± 0.1	28.6 ± 0.12	Carbon-centered	
	300/6	2.0042 ± 0.0002	6.8 ± 0.1	3.68 ± 0.01	Oxygen-centered	2
	400/6	2.0036 ± 0.0001	6.5 ± 0.2	7.35 ± 0.06	Carbon/Oxygen	
	500/6	2.0031 ± 0.0002	6.4 ± 0.1	13.3 ± 0.07	Carbon/Oxygen	
Corn straw	300/2	2.0040	4.786	3.966	Oxygen-centered	5
Corn stalks	500/2h	2.0049	6.9	9.67	Oxygen-centered	6
Maize straw	300/na	2.0037 ± 0.0001	6.8 ± 0.3	3.88 ± 0.08	Carbon/Oxygen	3
	400/na	2.0031 ± 0.0002	6.2 ± 0.2	6.25 ± 0.12	Carbon/Oxygen	
	500/na	2.0029 ± 0.0002	5.2 ± 0.1	30.2 ± 0.09	Carbon/Oxygen	
	300/6	2.0040 ± 0.0003	7.1 ± 0.3	1.25 ± 0.04	Oxygen-centered	2
	400/6	2.0038 ± 0.0002	6.9 ± 0.2	2.48 ± 0.11	Carbon/Oxygen	
	500/6	2.0035 ± 0.0001	6.4 ± 0.1	11.1 ± 0.05	Carbon/Oxygen	
Rice husk	300/na	2.0041 ± 0.0001	2.77 ± 0.05	6.9 ± 0.1	Oxygen-centered	7
	700/na	2.0036 ± 0.0001	0.16 ± 0.09	1.8 ± 0.1	Carbon/Oxygen	
	300/4	2.0039	6.9	2.77	Carbon/Oxygen	8
	400/4	2.0038	5.7	6.40	Carbon/Oxygen	
	500/4	2.0034	5.1	17.1	Carbon/Oxygen	
	600/4	2.0032	2.2	1.76	Carbon/Oxygen	
	700/4	2.0032	1.8	0.16	Carbon/Oxygen	
Bamboo	500/2h	2.0046	6.2	7.94	Oxygen-centered	6
Moso bamboo	300/2	2.0039		4.46	Carbon/Oxygen	9
	400/2	2.0032		6.89	Carbon/Oxygen	
	500/2	2.0026		24.5	Carbon-centered	
	600/2	2.0022		2.75	Carbon-centered	
Cellulose/urea (1/1)	400/30min	2.0040	5.3	1.634 ± 0.038	Oxygen-centered	10
	450/30min	2.0038	4.6	1.928 ± 0.042	Carbon/Oxygen	
	500/30min	2.0037	3.5	2.132 ± 0.039	Carbon/Oxygen	
	550/30min	2.0036	2.7	2.011 ± 0.043	Carbon/Oxygen	
	600/30min	2.0036	3.2	1.731 ± 0.024	Carbon/Oxygen	
**Non-lignocellulosic biomass**						
Sludge	200/2	2.0029	9.0	4.42 ($\times 10^{16}$)	Carbon-centered	11
	300/2	2.0035	8.0	4.26 ($\times 10^{16}$)	Carbon/Oxygen	
	400/2	2.0001	11.0	3.90 ($\times 10^{16}$)	Carbon-centered	

	500/2	2.0049	11.0	2.72 (×10^{16})	Oxygen-centered	
	600/2	2.0048	10.0	2.16 (×10^{16})	Oxygen-centered	
Laminaria japonica	200/2~4	2.0044	6.913	1.62	Oxygen-centered	12
	300/2~4	2.0039	8.405	40.2	Carbon/Oxygen	
	400/2~4	2.0035	8.015	42.4	Carbon/Oxygen	
	500/2~4	2.0029	3.568	46.1	Carbon-centered	
	600/2~4	2.0028	2.590	2.04	Carbon-centered	
	700/2~4	2.0029	6.110	0.220	Carbon-centered	
Pig manure	500/2h	2.0048	7.0	14.13	Oxygen-centered	6
Swine manure	300/2h	2.0044 ± 0.0002	6.8 ± 0.3	7.45 ± 0.03	Oxygen-centered	13
	600/2h	2.0041 ± 0.0001	7.0 ± 0.2	13.96 ± 0.04	Oxygen-centered	
	900/2h	2.0034 ± 0.0001	6.6 ± 0.3	11.23 ± 0.06	Carbon/Oxygen	
Cow manure	300/na	2.0046±0.0001	2.20 ± 0.01	7.1 ± 0.1	Oxygen-centered	7
	700/na	2.0036±0.0002	0.95 ± 0.06	3.4 ± 0.1	Carbon/Oxygen	

* Temp: Pyrolysis temperature/residence time (°C/h); ** Carbon/Oxygen: carbon-centered radicals with an adjacent oxygen atom (2.0030<g<2.0040) [Oxygen-centered (g>2.0040); Carbon: Carbon-centered (g<2.0030)]; *** na: not available (not mentioned).

Table S2. Main elements and atomic ratios of the resulting biochars

	N	C	H	Ash	O	H/C	O/C	Al	Ca	K	Mg	Na	Cu	Fe	Mn	Zn
	%	%	%	%	%			%	%	%	%	%	mg/kg	mg/kg	mg/kg	mg/kg
WC200	0.491	49.5	6.05	2.80	41.2	1.47	0.625	0.030	0.108	0.100	0.019	0.021	2.14	24.8	6.54	5.15
WC300	0.426	70.5	4.85	3.28	21.0	0.826	0.223	0.037	0.518	0.364	0.098	0.082	5.76	49.4	14.6	21.1
WC400	0.360	75.7	3.86	3.61	16.5	0.612	0.163	0.048	0.748	0.602	0.142	0.122	10.4	118	21.6	30.1
WC500	0.289	80.4	3.51	4.21	11.6	0.524	0.108	0.062	0.862	0.684	0.167	0.131	10.9	144	27.2	32.4
WC600	0.251	83.0	3.02	4.83	8.93	0.437	0.0807	0.071	0.894	0.753	0.183	0.132	11.2	346	37.5	33.2
WC700	0.120	84.7	2.41	5.09	7.68	0.341	0.0680	0.194	0.924	0.79	0.195	0.140	13.0	544	38.7	34.8
RH200	3.21	20.3	2.84	60.7	13.0	1.68	0.478	0.026	0.123	0.299	0.043	0.016	3.56	105	271	14.5
RH300	2.25	21.4	2.22	66.2	7.93	1.24	0.277	0.069	0.183	0.462	0.075	0.020	3.83	358	433	25.4
RH400	1.67	24.5	1.53	67.4	4.93	0.749	0.151	0.092	0.250	0.547	0.089	0.028	6.66	415	442	27.6
RH500	1.45	26.2	1.16	68.2	2.94	0.531	0.0841	0.109	0.289	0.583	0.091	0.035	5.60	480	456	32.4
RH600	1.12	27.4	0.94	68.8	1.82	0.412	0.0499	0.127	0.313	0.639	0.101	0.041	18.6	536	469	35.2
RH700	0.920	27.6	0.660	69.6	1.19	0.287	0.0323	0.169	0.329	0.663	0.110	0.054	17.7	720	490	43.5
PN200	0.950	39.7	4.95	22.8	31.5	1.50	0.596	0.064	0.754	0.413	0.344	0.025	4.71	93.2	846	44.8
PN300	0.780	43.8	4.16	30.2	21.1	1.14	0.362	0.094	0.936	0.957	0.621	0.044	12.7	173	1494	106
PN400	0.710	50.3	3.26	34.4	11.4	0.778	0.169	0.108	1.169	1.355	0.911	0.052	15.3	280	1833	171
PN500	0.510	52.8	2.50	35.52	8.71	0.569	0.124	0.117	1.429	1.673	1.051	0.062	22.6	333	2345	199
PN600	0.560	55.7	2.36	36.4	4.92	0.508	0.0662	0.120	1.456	1.775	1.153	0.075	24.0	381	2524	230
PN700	0.430	56.9	1.71	38.1	2.79	0.360	0.0367	0.128	1.505	1.948	1.161	0.093	32.0	417	2795	242
ADS200	3.83	57.9	6.16	3.62	28.5	1.28	0.368	6.975	6.46	0.559	0.346	0.750	878	29315	2658	2133
ADS300	3.33	66.1	5.26	5.46	19.8	0.954	0.225	7.962	7.489	0.595	0.389	0.844	1039	33485	3280	2542
ADS400	2.81	70.6	3.59	7.44	15.6	0.610	0.166	8.493	8.264	0.643	0.419	0.921	1126	37745	3941	2697
ADS500	2.43	76.5	3.15	8.19	9.71	0.494	0.0952	8.858	8.527	0.695	0.444	0.977	1183	38002	4032	2958
ADS600	1.08	77.8	2.57	9.94	8.62	0.396	0.0831	9.262	8.786	0.706	0.463	0.988	1202	38609	4157	3096
ADS700	0.950	79.9	2.33	12.9	3.97	0.350	0.0373	9.671	9.208	0.722	0.495	1.029	1262	38922	4394	3170
DW500	2.48	49.7	2.78	35.5	9.61	0.672	0.145	0.146	1.270	0.499	0.636	0.080	23.4	703	221	167
BK500	1.02	58.9	3.15	29.9	7.06	0.642	0.0900	0.025	0.904	0.122	0.134	0.020	19.8	361	217	91.1
CS500	0.730	71.1	3.37	14.0	10.9	0.569	0.115	0.114	0.545	0.447	0.560	0.078	21.8	341	152	104

Table S3. EPFRs in RH400 under different the pyrolysis time.

	Spin	ΔHp-p	g-factor
1h	5.53E+19	6.16	2.0029
2h	5.54E+19	6.06	2.0026
4h	6.19E+19	5.08	2.0028
8h	5.60E+19	8.49	2.0027

Table S4. Stability of EPFRs in RHx under room temperature.

	0 d			7th day			18th day		
	spins/g	g-factor	ΔHp-p	spins/g	g-factor	ΔHp-p	spins/g	g-factor	ΔHp-p
200°C	3.28E+17	2.0035	6.45	2.52E+17	2.0036	5.87	1.98E+17	2.0039	5.87
300°C	1.58E+19	2.0033	7.04	1.39E+19	2.0035	6.65	1.05E+19	2.0030	7.12
400°C	5.54E+19	2.0028	5.47	4.83E+19	2.0027	4.89	4.05E+19	2.0026	4.91
500°C	7.11E+19	2.0030	4.30	6.19E+19	2.0031	4.68	5.60E+19	2.0028	4.11
600°C	2.91E+19	2.0025	3.71	2.47E+19	2.0027	4.27	1.95E+19	2.0026	4.47
700°C	7.38E+15	2.0025	2.15	6.61E+15	2.0019	1.96	5.04E+15	2.0024	2.24

References

1 Y. Z. Zhang, M. Q. Xu, S. X. Liang, *et al.*, Mechanism of persulfate activation by biochar for the catalytic degradation of antibiotics: Synergistic effects of environmentally persistent free radicals and the defective structure of biochar, *Science of the Total Environment*, 2021, **794**, 148707.

2 G. D. Fang, C. Y. Zhu, D. D. Dionysiou, *et al.*, Mechanism of hydroxyl radical generation from biochar suspensions: Implications to diethyl phthalate degradation, *Bioresource Technology*, 2015, **176**, 210-217.

3 G. D. Fang, J. Gao, C. Liu, *et al.*, Key role of persistent free radicals in hydrogen peroxide activation by biochar: Implications to organic contaminant degradation, *Environmental Science & Technology*, 2014, **48**, 1902-1910.

4 Y. Z. Zhang, M. Q. Xu, X. K. Liu, *et al.*, Regulation of biochar mediated catalytic degradation of quinolone antibiotics: Important role of environmentally persistent free radicals, *Bioresource Technology*, 2021, **326**, 124780.

5 N. Zhao, Z. Yin, F. Liu, *et al.*, Environmentally persistent free radicals mediated removal of Cr(VI) from highly saline water by corn straw biochars, *Bioresource Technology*, 2018, **260**, 294-301.

6 D. Huang, H. Luo, C. Zhang, *et al.*, Nonnegligible role of biomass types and its compositions on the formation of persistent free radicals in biochar: Insight into the influences on Fenton-like process, *Chem. Eng. J.*, 2019, **361**, 353-363.

7 J. L. Qin, Y. X. Cheng, M. X. Sun, *et al.*, Catalytic degradation of the soil fumigant 1,3-dichloropropene in aqueous biochar slurry, *Science of the Total Environment*, 2016, **569**, 1-8.

8 J. L. Qin, Q. C. Chen, M. X. Sun, *et al.*, Pyrolysis temperature-induced changes in the catalytic characteristics of rice husk-derived biochar during 1,3-dichloropropene degradation, *Chem. Eng. J.*, 2017, **330**, 804-812.

9 Y. Zhang, M. Xu, R. He, *et al.*, Effect of pyrolysis temperature on the activated permonosulfate degradation of antibiotics in nitrogen and sulfur-doping biochar: Key role of environmentally persistent free radicals, *Chemosphere*, 2022, **294**, 133737.

10 D. Bi, F. Huang, M. Jiang, *et al.*, Effect of pyrolysis conditions on environmentally persistent free radicals (EPFRs) in biochar from co-pyrolysis of urea and cellulose, *Science of the Total Environment*, 2022, **805**, 150339.

11 Y. Hu, K. Tong, Q. Guo, *et al.*, Effect of controlled temperature and biomass addition on the formed environmental persistent free radicals (EPFRs) in sewage sludge-based biochar from pyrolysis treatment, *Journal of Analytical and Applied Pyrolysis*, 2022, **162**, 105460.

12 Y. Huang, X. Guo, Z. Ding, *et al.*, Environmentally persistent free radicals in biochar derived from *Laminaria japonica* grown in different habitats, *Journal of Analytical and Applied Pyrolysis*, 2020, **151**, 104941.

13 R. Deng, H. Luo, D. Huang, *et al.*, Biochar-mediated Fenton-like reaction for the degradation of sulfamethazine: Role of environmentally persistent free radicals, *Chemosphere*, 2020, **255**, 126975.
